# Immune signatures of megakaryocytes in persistent inflammation-immunosuppression and catabolism syndrome: Immune signatures of megakaryocytes in PICS

**DOI:** 10.3724/abbs.2025087

**Published:** 2025-05-30

**Authors:** Xingfeng Sun, Ke Nan, Ziwen Zhong, Zhiqiang Liu, Changhong Miao

**Affiliations:** 1 Department of Anesthesiology Zhongshan Hospital Fudan University Shanghai 200032 China; 2 Department of Anesthesiology Obstetrics and Gynecology Hospital of Fudan University Shanghai 200090 China

**Keywords:** persistent inflammation-immunosuppression and catabolism syndrome, sepsis, megakaryocyte, immune, single-cell RNA sequencing

## Abstract

Persistent inflammation-immunosuppression and catabolism syndrome (PICS) is a severe condition that may follow sepsis and is characterized by ongoing inflammation and immune suppression, diminishing quality of life and potentially causing death. The role of megakaryocytes (MKs) in PICS, despite their association with thrombopoiesis, is not well understood. In this study, we use single-cell RNA sequencing to profile MKs in peripheral blood mononuclear cell samples obtained from 11 patients, including six with PICS, five with sepsis, and five healthy controls, to determine the diversity and molecular signatures of the MKs. Five subgroups of MKs are identified (MK1–MK5), and their proportions vary across the groups. MK1 and MK2 are predominant in PICS. Gene Ontology analysis shows that genes related to antigen processing and presentation and IL-17 signaling are enriched in MK1, whereas genes associated with platelet degranulation and neutrophil activation are enriched in MK2. Moreover, the expression level of CCL5 is markedly increased in MKs. Ligand-receptor analysis reveals dynamic interactions among MKs and T cells, B cells, natural killer cells, monocytes, and macrophages, suggesting a broad role of MKs in immune homeostasis. In PICS model mice, MKs regulate systemic inflammation by reducing the levels of the proinflammatory cytokines TNF-α and IL-17A and promoting lung tissue repair. Our findings establish MKs as essential components of the immune system in PICS and provide new insights into their potential as therapeutic targets for post-sepsis immune dysfunction.

## Introduction

Sepsis, a major cause of morbidity and mortality worldwide, can lead to multiple organ dysfunction and long-term immune alterations [1]. Individuals who survive sepsis may experience “persistent inflammation-immunosuppression, and catabolism syndrome” (PICS), a condition characterized by metabolic derangements, immune dysfunction, and increased susceptibility to secondary infections [ 2– 4]. 

Although the precise mechanisms underlying PICS remain to be elucidated, elevated levels of C-reactive protein (CRP), myeloid-derived suppressor cells, and inflammatory cytokines, including interleukin (IL)-6 and IL-8, are associated with the pathogenesis of PICS [5]. Many previous studies have focused on the role of myeloid cells and lymphocytes in sepsis-induced immune suppression [ 1, 6]. However, recent evidence suggests that megakaryocytes (MKs), which are traditionally considered to function as platelet progenitors [ 7– 9], may contribute to immune regulation and inflammation in the PICS. MKs have been implicated in several inflammatory diseases, with roles in regulating neutrophil activation, modulating adaptive immunity, and contributing to the resolution of inflammation [ 10– 12]. However, the heterogeneity and functional dynamics of MKs in pathological states are underexplored, and the precise role of MKs in PICS remains unclear. 

Single-cell RNA sequencing (scRNA-seq) has revolutionized the characterization of cellular diversity, enabling the identification of novel immune cell subsets and their molecular signatures.

In the present study, we systematically characterized the MKs of patients with PICS, septic patients, and healthy individuals using single-cell transcriptomics. The role of MKs in the immune response during PICS secondary to sepsis was also assessed. Our findings revealed extensive MK reprogramming in the PICS and suggested that MKs play an immunoregulatory role in the PICS, potentially influencing post-sepsis immune homeostasis. Our results provide a comprehensive overview of the role of MKs in the immune landscape of patients with PICS following sepsis.

## Materials and Methods

### Study participants

Patients who were diagnosed with sepsis resulting from Gram bacterial infection were enrolled in this study from August 2021 to January 2022. Informed consent was secured from all participants or their authorized representatives. This study received approval from the Ethics Committee of Zhongshan Hospital, Fudan University (B2021-215R) and was registered with the Chinese Clinical Trial Registry (ChiCTR2100048148). Additionally, the animal experiments were authorized by the Department of Laboratory Animal Science at Fudan University (202108006s). The scRNA-seq data have been deposited in the Gene Expression Omnibus (GEO) database (GSE217906).

To be enrolled, patients needed to fulfil the diagnostic criteria for sepsis 3.0 [13]. Organ dysfunction was assessed using the Sequential Organ Failure Assessment (SOFA) score, and sepsis was identified by the presence of an infection in conjunction with a SOFA score indicating either a cardiovascular system function score of ≥ 1 or a score of ≥ 2 in any other organ system [ 14– 16]. Individuals identified with PICS had to meet the following conditions: an ICU ≥ 14 days; CRP levels greater than 0.15 mg/dL; serum albumin (ALB) levels less than 30 g/L; prealbumin (PA) levels under 10 mg/L; retinol-binding protein (RPB) levels below 10 mg/L; a total lymphocyte (LYM) count below 0.8 × 10 ^9^/dL; a creatinine height index (CHI) of less than 80%; and either a weight loss exceeding 10% or a body mass index (BMI) below 18 during their hospitalization [ 2, 3]. The control group comprised healthy volunteers. The exclusion criteria included individuals with immunodeficiency and those receiving treatment with immunomodulatory medications, such as steroid analogues, prednisone, or biological immune system modulators. Sepsis was defined as sepsis diagnosed within 24 h. 

### Peripheral blood mononuclear cell (PBMC) collection

PBMCs were extracted from blood samples using density gradient centrifugation, as previously described in detail [17]. In brief, whole blood was diluted at a 1:1 ratio with 1 × phosphate-buffered saline (PBS), carefully layered over Ficoll-Paque Plus (Cytiva, Marlborough, USA), and subjected to centrifugation at 400 *g* for 30 min. The PBMC layer was subsequently collected and washed twice with Dulbecco’s PBS at 300 *g* for 10 min. Erythrocytes were removed using red blood cell lysis on ice. The cells were resuspended in RPMI 1640 medium (HyClone, South Logan, USA) supplemented with 10% fetal bovine serum (FBS; HyClone) following centrifugation. Cell viability was assessed using trypan blue exclusion and was determined to be approximately 90% for each sample. 

### Single-cell preparation

The scRNA-seq experiments were conducted by NovelBio (Shanghai, China). Blood samples were prepared by mixing with anticoagulant and then measured and diluted with PBS. Once the blood was fully added to the separation tube, it was stratified with the separation solution. The samples were subsequently centrifuged at 800 *g* for 20 min to reduce vibration. Lymphocytes were carefully extracted from the middle layer using a pipette, transferred to a new 1.5 mL tube, and then centrifuged at 300 *g* for 10 min. Finally, the liquid was collected with a 200 μL pipette, avoiding the formation of a precipitate. 

The precipitated cells were resuspended in red blood cell lysis buffer (Milton Biotec, Bergisch Gladbach, Germany) and then washed with PBS containing 0.04% bovine serum albumin (BSA; Sigma-Aldrich, St Louis, USA). The cell pellet was resuspended in PBS-BSA, filtered through a 35-μm filter, and single cells were stained with acridine orange/propidium iodide for viability assessment using a CountStar fluorescence cell analyzer (Alit Biotech Co., Ltd., Shanghai, China).

### scRNA-seq and statistical analysis of single-cell RNA data

As instructed by the manufacturer, the cells were labelled with TotalSeqTM-C antibodies. The samples were subsequently resuspended in 50 μL of BioLegend cell staining buffer (BioLegend, San Diego, USA), tagged, and incubated for 30 min at 4°C. After 3.5 mL of buffer was added, the samples were centrifuged at 400 *g* for 5 min, washed twice, resuspended, counted, and pooled in PBS. scRNA-seq and V(D)J libraries were generated using the 10 × Genomics Chromium platform (NovelBio). After reverse transcription, the GEMs were disrupted, and barcoded cDNA was purified and amplified to increase 5′ gene expression. The Qubit high-sensitivity DNA assay was used to quantify the libraries, and their size distribution was analyzed using a Bioanalyzer (Agilent, Santa Clara, USA). Sequencing was performed with 150 bp paired-end runs on an Illumina sequencer (San Diego, USA). 

The scRNA-seq data were analyzed by NovelBio using the NovelBrain Cloud Analysis Platform ( www.novelbrain.com). Using fastp [18] with default settings, we filtered out adaptor sequences and removed low-quality reads to acquire clean data. The matrices of feature barcodes were created by mapping reads to the human genome (GRCh38 Ensemble: version 91) using CellRanger v3.1.0. We filtered for quality by selecting cells with more than 200 expressed genes and mitochondrial UMI rates under 20%, excluding mitochondrial genes from the expression table. 

The Seurat package (v3.1.4; https://satijalab.org/seurat/) was utilized for cell normalization and regression using UMI counts and mitochondrial percentages to obtain scaled data. Principal component analysis was conducted on the top 2000 highly variable genes, with the first 10 components used for t-distributed Stochastic Neighbor Embedding (tSNE) construction. Unsupervised cell clusters were identified using a graph-based method, and marker genes were calculated with the FindAllMarkers function, applying criteria of ln(Fold Change) > 0.25, *P* value < 0.05, and min.pct > 0.1. Clusters were further sub-clustered and analyzed for markers to identify different cell types more precisely. 

### Gene ontology (GO) and pathway enrichment analysis

A GO enrichment analysis was conducted to clarify the biological significance of marker genes and differentially expressed genes (DEGs) [19]. GO annotations were sourced from the Gene Ontology databases ( http://www.geneontology.org/), National Center for Biotechnology Information (NCBI; http://www.ncbi.nlm.nih.gov/), and UniProt ( http://www.uniprot.org/). Fisher’s exact test identified significant GO categories, with *P* values corrected for the false discovery rate. Using the Kyoto Encyclopedia of Genes and Genomes (KEGG) database, pathway analysis revealed important functional pathways associated with marker genes and DEGs. Fisher’s exact test identified pathways that were significantly enriched [20], with a *P* value < 0.05 indicating significance. 

### Cell communication analysis

We conducted a comprehensive study of cell-cell communication using CellPhoneDB [21], a public database of ligands, receptors, and their interactions. We characterized the cluster’s membrane, secreted proteins, and peripheral proteins across various pseudo-time intervals and calculated significant mean values and cell communication metrics ( *P* < 0.05) by employing interaction data and normalized cell matrices derived from Seurat normalization. 

### DEG analysis

To detect DEGs, the Seurat FindMarkers function was used with the Wilcoxon rank-sum test, applying criteria such as ln (Fold Change) > 0.25, *P* value < 0.05, and min.pct > 0.1. To identify DEGs, the FindMarkers function in Seurat was employed via the Wilcoxon rank-sum test algorithm. The criteria applied were ln(Fold Change) > 0.25, *P* value < 0.05, and a minimum percentage (min.pct) greater than 0.1. 

### Mice

Male C57BL/6 mice were procured from Jihui Laboratory Animal Care (Shanghai, China). The animals were maintained under standardized environmental conditions and were given unrestricted access to a commercial pellet diet and water.

### Animal experiments and cecal ligation and puncture

The mice used in the experiments were aged 8–10 weeks (20–25 g). Cecal ligand and puncture (CLP) surgeries were performed as described previously [ 22, 23], from 9 a.m. to 12 p.m. Briefly, the mice were quickly anaesthetized via an intraperitoneal (i.p.) injection of 2.0% pentobarbital at a concentration of 80 μg/mg. Polymicrobial sepsis was induced through 33% cecal ligation and a single 25-gauge needle puncture [ 24, 25]. The bowel contents were removed from the puncture site, and the incision was closed with a 3-0 silk suture. Post-CLP, the mice received 1 mL of saline via i.p. injection and were allowed to recover on a 37°C electric blanket for 60 min. CLP mice were intraperitoneally injected with antibiotics [14 mg/kg primaxin (imipenem and cilastatin); 00006-3516-59; Merck, Whitehouse Station, USA] after closure of the incision. Meloxicam (5 mg/kg; product number: M3935; Sigma-Aldrich) was given subcutaneously after surgery for pain relief. Cohort sizes were based on prior data considering variability and mortality after moderate CLP injury [23]. 

### PICS mouse model

Surviving mice displayed PICS symptoms, including elevated myeloid cells, decreased lymphocytes, and weight loss, eight days following CLP [ 26– 28]. These mice were used as PICS models in the experiments. The control groups included untouched and sham-surgery mice, which presented similar inflammation and coagulation levels at 2 and 8 days post-surgery [27]. 

### Harvesting of the spleen, bone marrow, and blood cells and determination of cell counts

Spleens and bone marrow from septic, PICS, and sham mice were homogenized in PBS and filtered through a 70-μm strainer to generate a single-cell suspension. Cell counts were determined using a Beckman Coulter counter (Beckman Coulter, Pasadena, USA). Whole blood was drawn via cardiac puncture into EDTA K2 tubes. The cells (1–5 × 10 ^6^) were then analyzed by flow cytometry. 

### Flow cytometry

Cell surface antigens were examined via flow cytometry using a CytoFLEX (Beckman Coulter). The cells were incubated in 1 μL of FBS staining buffer with Fc Block (BD Pharmingen, San Jose, USA). Viability was determined by excluding cells stained with the live/dead fixable viability dye EF780 (Thermo Fisher Scientific, San Diego, USA). The following antibodies obtained from BD Biosciences (San Jose, USA) were used: CD45 (clone 30-F11), CD4 (clone GK1.5), CD8 (clone 53-6.7), CD62L (MEL-14), F4/80 (clone T45-2342), LY6G (clone 1A8), LY6C (clone HK1.4), CD41 (clone MWReg30), CD61 (clone 2C9. G2), CD19 (clone HIB19), CD138 (clone DL-101), CD25 (clone 2A3), and FOXP3 (clone MF23).

### Isolation of blood MKs

Blood was collected from septic and PICS mice through cardiac puncture into EDTA K2 tubes (BD Biosciences). The CD41 ^+^ cells were subsequently identified as MKs [29]. The Megakaryocyte Isolation Solution kit (Haoyang Biological Co., Ltd., Tianjin, China), MinIMACS Starting Kit, and anti-FITC MicroBeads (Miltenyi Biotec, Bergisch Gladbach, Germany) were used to isolate MKs according to the manufacturer’s guidelines. MKs were set to a concentration of 1.0 × 10 ^6^ cells/mL and counted with a Beckman Coulter cell counter (Beckman Coulter). 

### MK adoptive transfer assay and thrombopoietin (TPO) assay

The mice were categorized into four groups: sepsis (2 days after CLP), sepsis control (sham surgery, 2 days after operation), PICS (8 days after CLP), and PICS control (sham surgery, 8 days after operation) groups. Half of the mice were administered with MKs (0.2 mL, 2.0 × 10 ^5^ cells per mouse) via injection through the tail vein or with thrombopoietin (TPO) (12 μg/kg; batch number: P40226; Absin Bioscience, Shanghai, China) [30] via subcutaneous injection, while the other half received PBS (0.2 mL) only. The sepsis groups received MKs from septic mice on days 2, 3, and 4, whereas the PICS groups received MKs from PICS mice on days 8, 9, and 10. The TPO regimen is the same as the injection of MK. The samples were collected two days after the intervention. 

### Hematoxylin and eosin (H&E) staining

Lung tissue from septic, PICS, and control mice was harvested and preserved in 10% formalin following H&E staining. Microscopic analysis was conducted using an Imager M2 microscope (Zeiss, Thornwood, USA), with images captured at consistent exposure times for each magnification level. The extent of tissue damage and the number of microthrombi per organ field were quantified by two independent, blinded observers.

### *In vivo* cell depletion 

To deplete specific cell types, PICS mice were injected intraperitoneally (i.p.) with anti-mouse CSF1R (CD115) (clone AFS98, which is usually used as a marker of bone marrow cells, such as monocytes, macrophages, and DCs) [ 31 – 33], anti-mouse B220 (clone AFS98, anti-B cell) [34], or anti-mouse CD25 (clone AFS98, anti-Treg) [35] monoclonal antibodies (BioXCell, West Lebanon, USA). The treatment regimen was structured as follows: 250 μg of each antibody in 0.2 mL of PBS was injected i.p. into each PICS mouse on days 1 and 4 and into each septic mouse on day 1. The control (sham) mice were injected with 0.2 mL of PBS at the same time points. 

### Cytokine measurement

After the samples were centrifuged at 3000 *g* at 4°C for 20 min, the serum was extracted and preserved at –80°C. Serum cytokine levels were assessed using the Luminex200 platform according to the manufacturer’s protocol (LabEx; Uniway Biotech, Shanghai, China). 

### Statistical analysis

All the statistical analyses were conducted via GraphPad Prism 8 software (La Jolla, USA). Group comparisons were performed via Student’s *t* test, whereas one-way analysis of variance (ANOVA) was used for multiple group comparisons. Data are presented as the mean ± standard deviation (SD), with a statistical significance level of *P* < 0.05. 

## Results

### scRNA-seq maps of the MK signature in PICS

**Figure 1:**
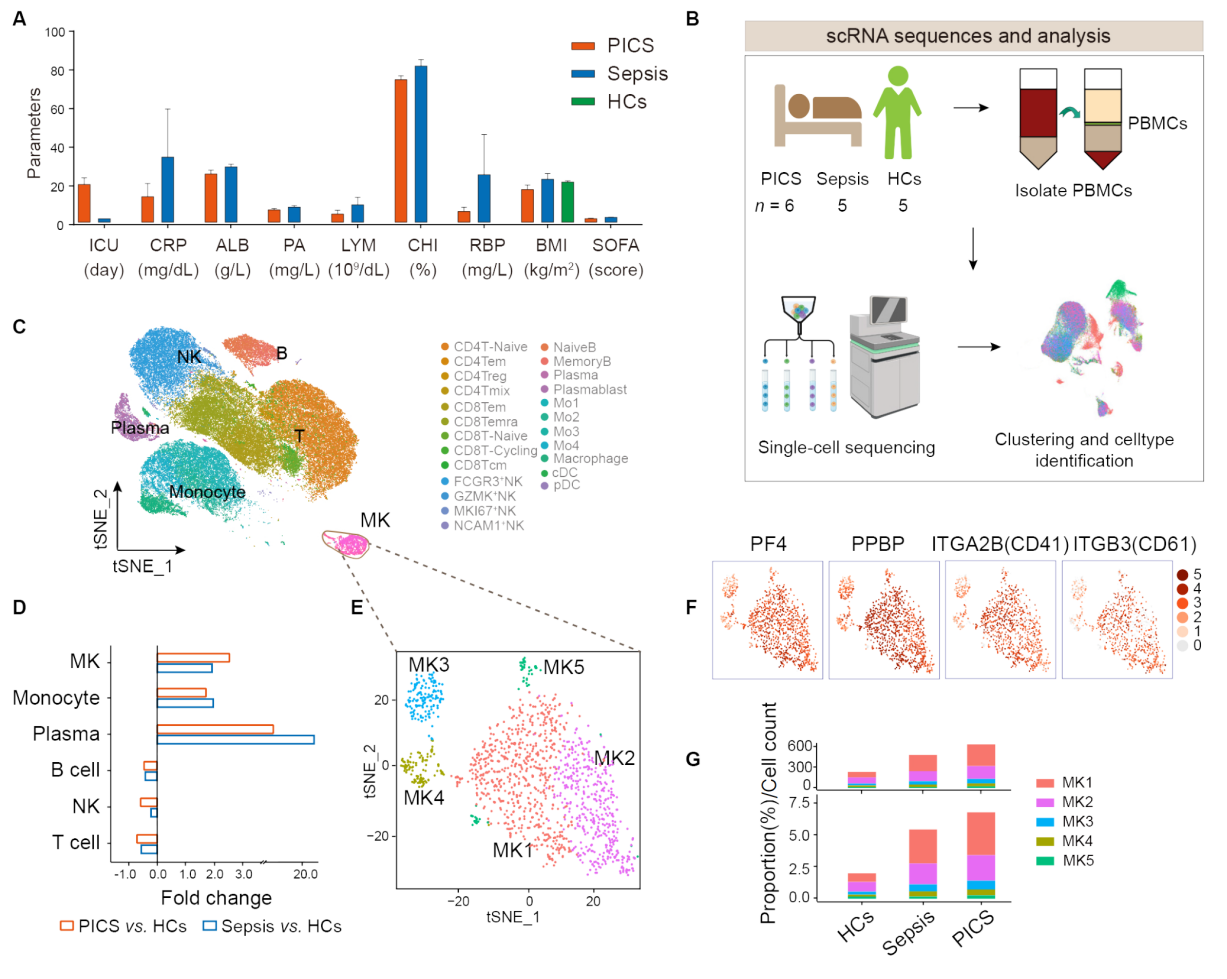
Figure 1 Study design and characterization of MKs from study subjects at the single-cell level (A) Histogram of laboratory examination results of participants. (B) Study design and schematic workflow. (C) tSNE plot of 92,912 high-quality single cells generated from equal subsampling by subset. (D) Differential cell types were identified through fold change filtering. (E) tSNE plot of the MK subclusters. (F) tSNE plot of the MK marker genes PF4, PPBP, IRGA2B (CD41), and ITGB3 (CD61). (G) Histogram of the MK subsets in patients with PICS or sepsis and HCs.

To investigate the cellular diversity and molecular characteristics of MKs in patients with PICS, PBMCs were collected from a cohort of 11 individuals, comprising six patients diagnosed with PICS, five patients with sepsis, and five healthy control subjects. All patient laboratory results fulfilled the diagnostic criteria ( Figure 1A). The PBMCs were subjected to scRNA-seq ( Figure 1B). Rigorous quality control protocols were used to confirm that the data were derived from single, viable cells (92,912 cells). Through principal component analysis and tSNE dimensionality reduction visualization, PBMCs were classified as T cells (CD4 T-Naïve, CD4 Tem, CD4 Treg, CD4 Tmix; CD8 Tem, CD8 Temra, CD8 T-Naïve, CD8 T-Cycling, and CD8 Tcm), natural killer cells (NK cells; FCGR3 ^+^NK, GZMK ^+^NK, MKI67 ^+^NK, and NCAM ^+^NK), B cells (naïve and memory B cells), plasma cells (plasma and plasmablast cells), monocytes (Mo1-4 and macrophages), MKs, conventional dendritic cells (cDCs) and plasmacytoid DCs ( Figure 1C and Supplementary Table S1). No significant batch effects were detected.

Among these immune cell populations, the increase in MKs was more pronounced in patients with PICS and sepsis than in HCs ( Figure 1D). Next, on the basis of the presence of the MK marker genes platelet factor 4 ( *PF4* ), *PPBP*, *IRGA2B* ( *CD41*), and *ITGB3* ( *CD61*), we captured and grouped 1379 MKs and identified five subgroups of MKs: MK1-MK5 ( Figure 1E,F and Supplementary Table S1). Intriguingly, the number of MKs expanded markedly in patients with PICS and sepsis, and the level of expansion was greater in those with PICS than in those with sepsis, especially in the MK1 and MK2 subsets ( Figure 1G), indicating notable shifts in the peripheral immune system during PICS and sepsis. 

### MKs show anti-inflammatory and immunoregulatory functions

**Figure 2:**
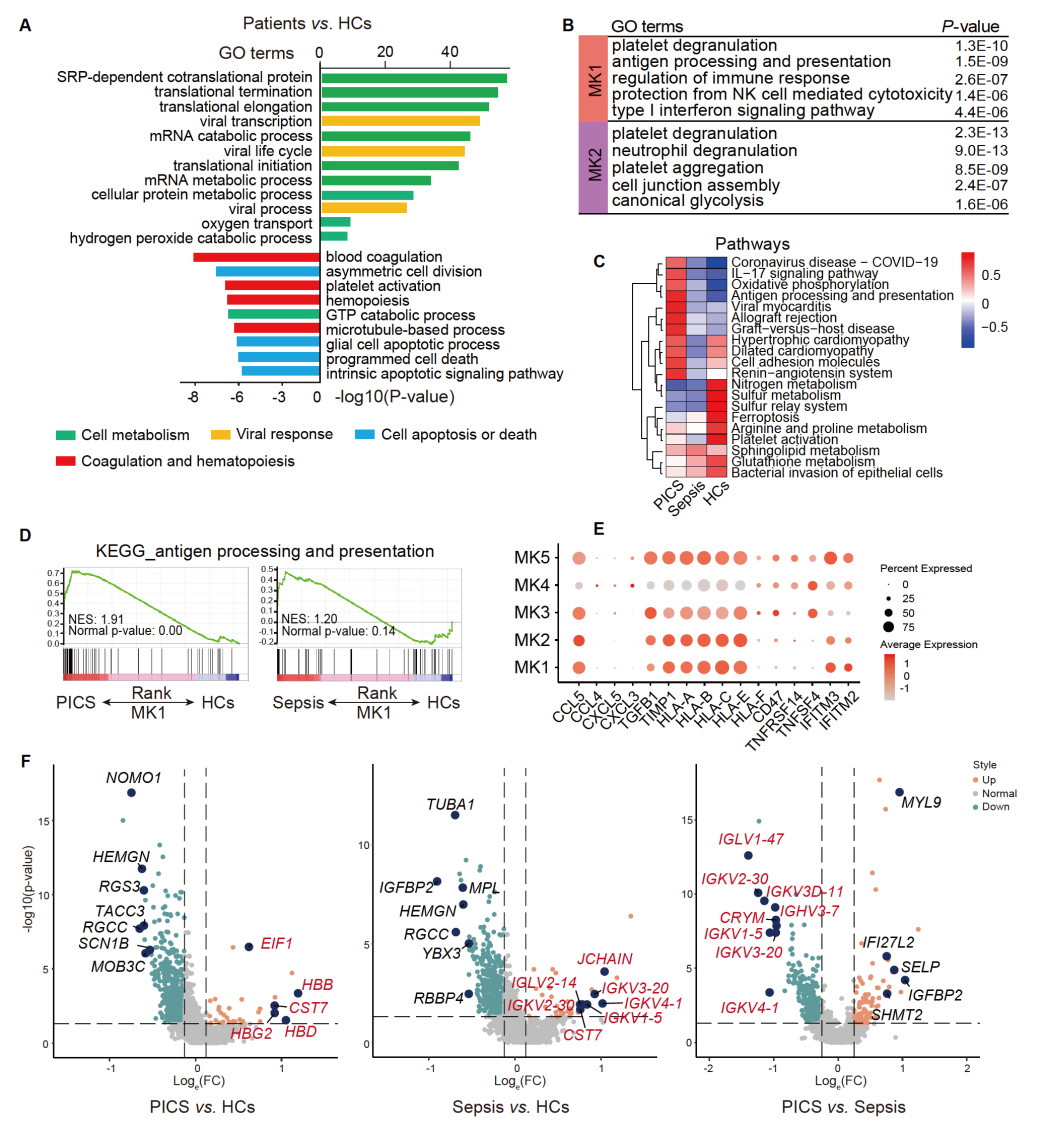
Figure 2 MKs function in anti-inflammatory and immune regulatory responses (A) Histogram of enriched gene ontology (GO) terms in MKs between patients with PICS or sepsis and HCs. (B) Enriched GO terms in the MK1 and MK2 subsets. (C) Heatmap of enriched GO pathways in the MKs of patients with PICS or sepsis and HCs. (D) Plot of the enriched candidate pathways related to antigen processing and presentation pathways in MK1 identified via gene set enrichment analysis. (E) Bubble plot of the activating and inhibitory ligand genes (chemokines, growth factors, immune checkpoints) expressed by the MK subsets. (F) Volcano plot of the differentially expressed genes identified via the two-sided Wilcoxon rank-sum test. Genes with ln(Fold Change) > 1 are shown in orange or cyan, and the top 10 genes with the highest positive fold changes are shown in blue.

The evidence suggests that MKs function in various biological processes, including coagulation, hemostasis, inflammation, and the immune response [ 7, 8, 36]. To clarify the role of MKs in PICS and sepsis, we analyzed the function of MKs through GO enrichment analysis. The analysis revealed multiple functional changes in MKs between patients with PICS and those with sepsis and HCs ( Figure 2A). GO terms related to MK substance synthesis, metabolism, protein translation, and antiviral infection were enriched in the PICS and sepsis (green) groups compared with the HCs, indicating that MKs were activated in these disease states and that their antiviral functions were active (yellow). In contrast, GO terms related to apoptosis and cell death were downregulated in MKs, indicating a state of enhanced survival or supporting a state of quantitative expansion (blue). The observed decrease in coagulation-associated features (red) may therefore be attributed to the predominance of the MK subpopulations, which exhibit immune-related characteristics and may represent immune MKs mobilized from the bone marrow rather than those induced by the peripheral immune microenvironment [ 37, 38]. Notably, the MK1 and MK2 subgroups appear to have different biological functions on the basis of gene expression. The MK1 subgroup showed specific enrichment of several immune-related gene sets, such as genes related to antigen processing and presentation, regulation of the immune response, protection from NK cell-mediated cytotoxicity, and the type I interferon signaling pathway ( Figure 2B), indicating that the MK1 subgroup primarily regulates innate and adaptive immune functions. MK2 cells were enriched in genes related to platelet degranulation, neutrophil degranulation, and platelet aggregation ( Figure 2B), indicating their association with blood coagulation and enhanced neutrophil-killing functions and suggesting that the MK2 subset is not only involved in the innate immune response but also contributes to the pathogenesis of thrombotic disorders. Interestingly, in patients with PICS and sepsis, the MK1 and MK2 subsets accounted for approximately 75% of the total MK cell count ( Figure 1F), which is much greater than the 7.1% in normal bone marrow [7], indicating that during infection, MKs tend to differentiate toward subtypes with immunomodulatory functions. In addition, in patients with PICS, the analysis revealed marked enrichment of GO pathway terms involved in immune responses related to coronavirus disease, the IL-17 signaling pathway, and antigen processing and presentation and downregulation of the ferroptosis pathway ( Figure 2C). Similarly, gene set enrichment analyses revealed that genes related to antigen processing and presentation in MK1 cells were enriched in patients with PICS compared with HCs ( Figure 2D).

The MKs also expressed chemokine genes ( *CCL5*, *CCL3*, *CXCL5*, and *CXCL3*), growth factor genes ( *TGFB1* and *TIMP1*), NK cell-inhibitory ligands involved in immune tolerance ( *HLA-A*, *HLA-B*, and *HLA-C*), immune checkpoint inhibitory ligand genes ( *HLA-E*, *HLA-F*, and *CD47*), immune checkpoint-activating ligand genes ( *TNFSF4*), bidirectional immune co-regulatory molecule ( *TNFRSF14*) and antiviral genes ( *IFITM3* and *IFITM2*) ( Figure 2E). These transcriptomic data suggest that MKs may contribute to anti-inflammatory and immunoregulatory processes through the expressions of chemokines, cytokines, and immune modulatory surface molecules. 

Analysis of the upregulated and downregulated DEGs revealed that the immune regulatory gene *CST7* was highly expressed in both patients with PICS and those with sepsis. Genes related to the clonal expansion and differentiation of B cells into immunoglobulin-secreting cells ( *JCHAIN* and the *IGKV* family) were upregulated in patients with sepsis ( Figure 2F), indicating that MKs participate in immune response regulation. Moreover, compared with that in patients with sepsis, the expressions of *IGKV* family genes were decreased in individuals with PICS ( Figure 2F), indicating that the involvement of MKs in humoral immune regulation is reduced in patients with PICS, which may be related to the decreased degree of inflammation in patients with PICS compared with that in septic patients. 

### Cell-cell communication dynamics in MKs in PICS

**Figure 3:**
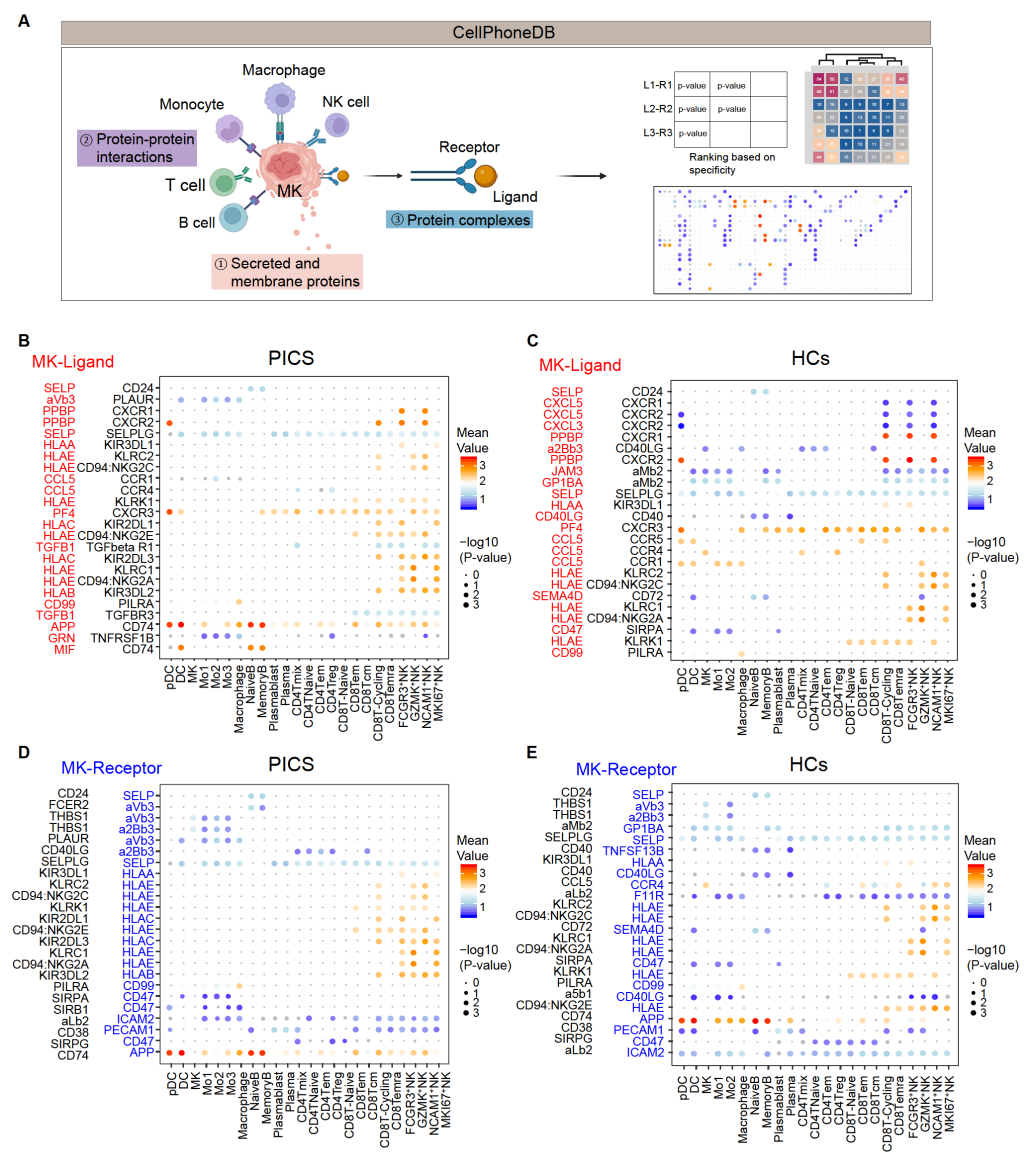
Figure 3 Cell-cell interactions in PBMCs identified through ligand-receptor analysis (A) Schematic diagram of the CellPhoneDB algorithm. (B–E) Detailed view of selected ligand-receptor interactions between MKs and other cell types with (B) MK ligands in PICS, (C) MK ligands in HCs, (D) MK receptors in PICS, and (E) MK receptors in HCs. Red font, MK ligands; blue font, MK receptors.

We studied the intercellular communication between different cell populations to explore the mutual regulation of human PBMCs. Using the CellPhoneDB algorithm, we systematically examined the interactions between ligands and receptors across every cell type [39] ( Figure 3A). Our analyses identified and counted the number of junctions between each cell type pair. In the PICS and HC groups, the cell types with greater numbers of intercellular connections were NK cells, T cells, monocytes, and macrophages, followed by B cells and plasma cells ( Figure 3B–D).

We next investigated the interactions between increased numbers of MKs and other cell types and their possible functions. Interestingly, MK ligands interact with other cell receptors, primarily through PPBP-CXCR1, PPBP-CXCR2, SELP-CD24, CCL5-CCR1, CCL5-CCR4, PF4-CXCR3, APP-CD74, and the HLA family (HLAA, HLAB, HLAC, and HLAE), particularly in patients with PICS ( Figure 3B,C). Specifically, monocyte-macrophage-chemotaxis is induced by CCL5 [ 40 , 41], whereas PPBP-CXCR1-2 induces chemotaxis [42] in NK and NKT cells. The PF4-CXCR3 interaction induces chemotaxis in various cell types [43]. Notably, the TGFB1 ligand was expressed only in patients with PICS ( Figure 3B,C), suggesting that MK regulates immune system function by increasing the expression of TGFB1, which is increased in these patients compared with HCs. The interaction between MKs, Mo1-3, and macrophages was manifested mainly through receptors associated with particle formation MK receptors (aVb3 complex, a2Bb3 complex, and aMb2 complex), CD47, and APP in patients with PICS and HCs. Interactions between MKs and B cells via CD24-SELP promote cell migration [44]. Together, there is extensive intercellular communication between MKs and other immune cells, which is enhanced in PICS. 

### Enriched population percentage of MKs in PICS model mice

**Figure 4:**
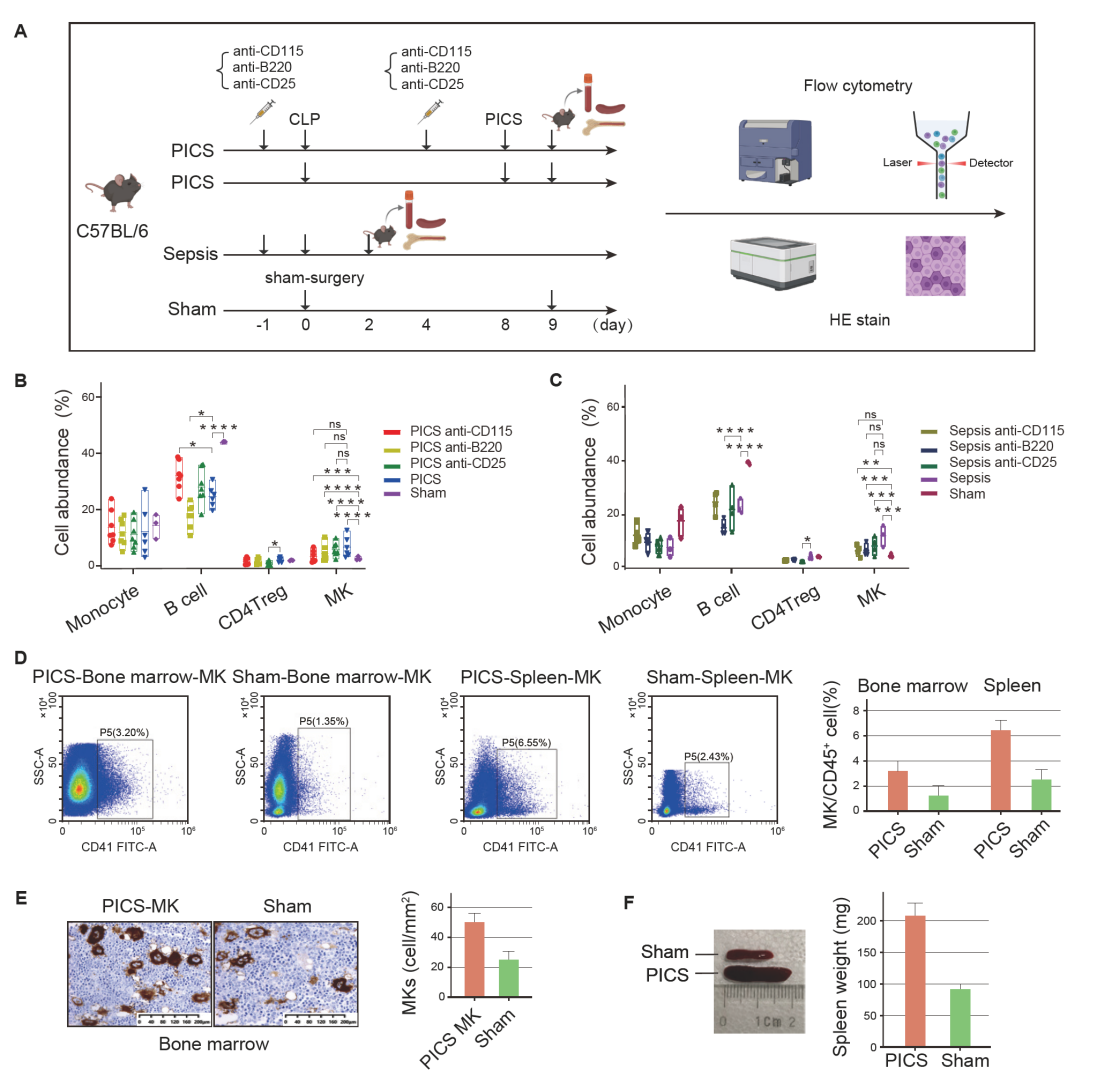
Figure 4 MKs in the peripheral blood, bone marrow, and spleen of PICS and sepsis model mice and control sham mice (A) Schematic diagram showing the timeline of the experimental design. (B,C) Proportions of monocytes, B cells, plasma cells, and MKs in (B) PICS and (C) sepsis model mice after injection with anti-CD115, anti-B220, or anti-CD25 antibodies (n = 5–6 mice per group). *P < 0.05, **P < 0.01, ***P < 0.001, ****P < 0.0001; ns, not significant by one-way ANOVA. The error bars represent the range of the changes. (D) The proportions of MKs in the bone marrow and spleen of PICS model mice and sham mice. (E) CD41 staining of MKs in the bone marrow of PICS model mice and sham mice. (F) Size of two representative spleens from sham and PICS model mice. The error bars represent the SDs.

There are extensive interactions between MKs and other types of immune cells. However, whether the depletion of immune cells affects the number of MKs is unclear. To explore this, we intraperitoneally (i.p.) injected PICS and sepsis model mice with anti-CD115, anti-B220, and anti-CD25 antibodies to deplete monocytes, B cells, and CD4 Tregs, respectively, and then observed whether there was a change in the number of MKs ( Figure 4A). After antibody injection, there were no significant changes in the proportions of monocytes, but the proportions of B cells and CD4 Tregs were significantly reduced ( Figure 4B,C), indicating that injection of the anti-CD115 antibody was ineffective, whereas injection of the anti-B220 and anti-CD25 antibodies was effective. MK cells expanded in four PICS subgroups ( *i*. *e*., the anti-CD115, anti-B220, anti-CD25, and control-injected PICS groups) and four sepsis subgroups ( *i*. *e*., the anti-CD115, anti-B220, anti-CD25, and control-injected sepsis groups) ( Figure 4B,C). Although B cells and CD4 ^+^ Tregs were efficiently depleted in PICS mice, the relative abundance of MKs in the anti-CD115, anti-B220, and anti-CD25 groups did not differ significantly from that in untreated PICS mice, suggesting that MK expansion occurs independently of these immune cell populations ( Figure 4B). The same results were obtained in the sepsis groups ( Figure 4C), indicating that MKs were not affected by changes in the numbers of B cells and CD4 Tregs.

MKs were enriched in the peripheral blood, bone marrow, and spleen of PICS mice compared with those of sham mice ( Figure 4D–F), suggesting the expansion or mobilization of MKs under PICS conditions. However, whether this enrichment reflects increased proliferation requires further investigation. 

### MKs exert anti-inflammatory and immunomodulatory effects in PICS mice

**Figure 5:**
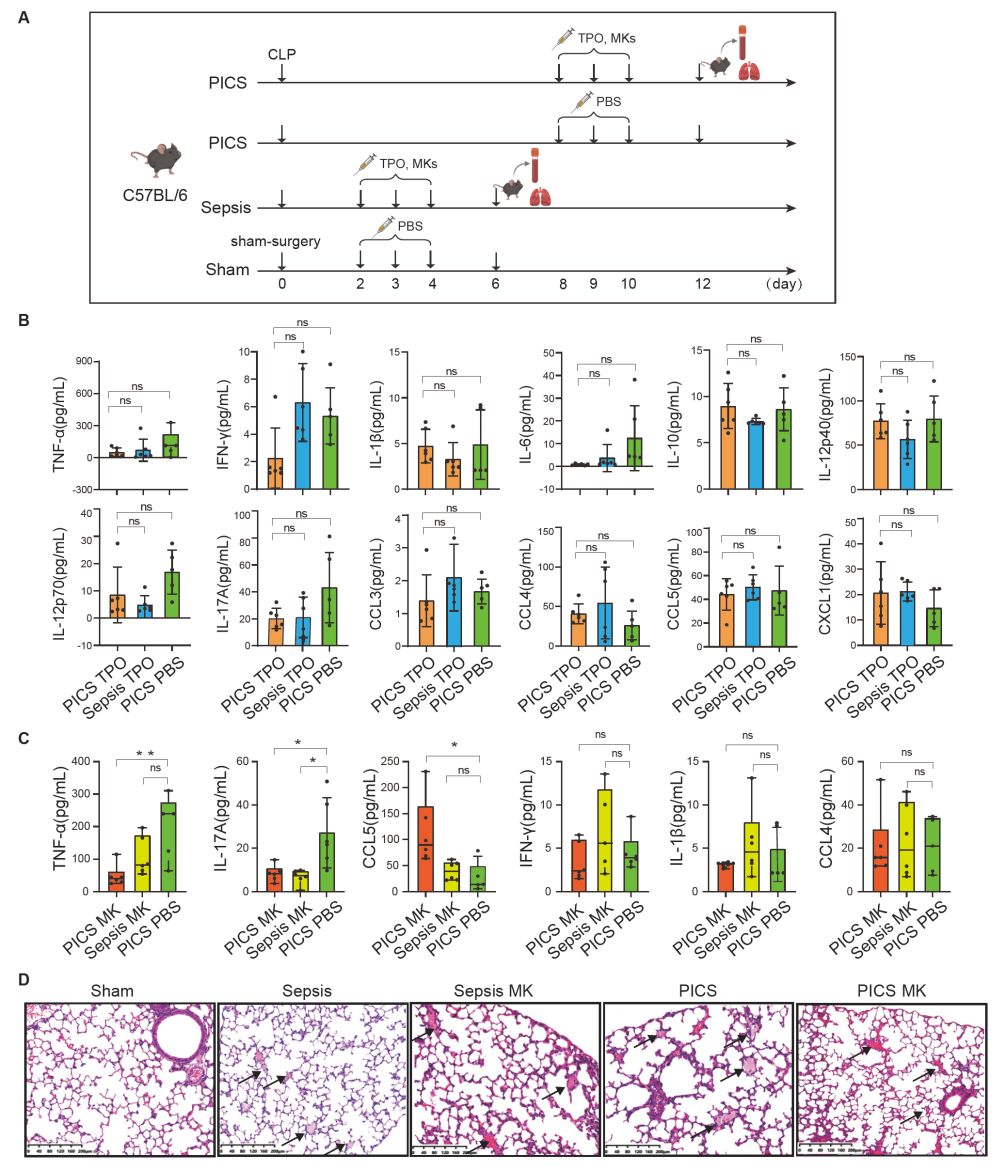
Figure 5 Effect of MK transfer on chemokine production and lung damage in PICS and sepsis model mice (A) Schematic diagram of the timeline of the experimental design. (B,C) Changes in the expression levels of inflammatory factors and chemokines in PICS and septic mice treated with (A) TPO (by i.p.) or (B) MKs (by i.v.) (n = 5–6 mice per group). *P < 0.05, **P < 0.01, ***P < 0.001, ****P < 0.0001; ns, not significant by one-way ANOVA. The error bars represent the range of the changes. (D) H&E staining of lung tissue (10×, magnification) from the sham, PICS, and sepsis groups with and without MK treatment. The black arrows indicate transparent thrombi.

Recent studies have reported that MKs secrete a variety of cytokines [ 11, 45]. TPO is a core factor that regulates megakaryogenesis and platelet production, and its effects run through the entire life cycle of MKs. We treated PICS and sepsis model mice with MKs or TPO and evaluated subsequent changes in systemic cytokine levels to determine whether MKs contribute to modulating the inflammatory response *in vivo* ( Figure 5A). The results revealed that after the injection of TPO, there was no significant change in the levels of the other tested cytokines (TNF-α, IFN-γ, IL-β, IL-6, IL-10, IL-12p40, IL-12p70, IL-17A, CCL3, CCL4, CCL5, and CXCL1; Figure 5B). Importantly, we conducted MK transfer experiments in mice to study the effects of MKs on cytokine levels during PICS and sepsis ( Figure 5A). Compared with those in PBS-injected mice, the serum levels of the proinflammatory factors TNF-α and IL-17A were significantly lower after the injection of MKs into the tail vein of PICS and sepsis model mice, whereas the serum levels of the chemokine CCL5 were increased **(** Figure 5C), suggesting that MKs at different stages of maturity ( *i*. *e*., in septic mice at 2 d after CLP and in PICS mice at 8 d after CLP) may function differently. Taken together, these results indicate that MKs from individuals with PICS or sepsis are involved in anti-inflammatory and immunomodulatory reactions, which is consistent with the single-cell RNA sequencing results.

MKs are located in the pulmonary microcirculation and extravascular space (interstitium) of the lungs and migrate in significant numbers from the bone marrow to the lungs at a rate of several hundred thousand to over one million per hour [ 46, 47]. To investigate whether the injection of MKs into PICS and septic mice has a lung-protective effect, we performed H&E staining of the lungs post-injection. We observed that the alveolar walls were damaged to varying degrees and that many transparent thrombi formed in the lungs of the groups of mice with disease, especially the PICS group ( Figure 5D). There was no decrease in the number of transparent thrombi in sepsis and PICS model mice after MK injection. Interestingly, the alveolar walls in the PICS mice were repaired after MK injection, indicating that MKs conferred a certain degree of protection to the lungs ( Figure 5 D).

## Discussion

This study systematically characterized the MKs of patients with PICS and sepsis using scRNA-seq and identified five functional subgroups (MK1-MK5). Among them, MK1, which highly expresses antigen-presenting genes, and MK2, which is enriched in coagulation-related genes, were significantly expanded in patients with PICS and sepsis. Notably, experiments revealed that transferred MKs reduced the levels of proinflammatory cytokines such as TNF-α and IL-17A and improved alveolar repair in injured lungs, highlighting their anti-inflammatory and tissue-protective potential. Collectively, these results suggest that MKs are pivotal regulators of immune homeostasis in PICS and sepsis and offer novel therapeutic targets for mitigating systemic inflammation and organ damage.

Despite accumulating knowledge, the mechanisms underlying the functional specialization of MK subpopulations remain poorly understood. Recent studies suggest that MKs originating from distinct hematopoietic trajectories may play divergent biological roles, with those following a myeloid differentiation pathway acquiring immune-like phenotypes [48]. Additionally, environmental cues and anatomical localization within bone marrow or extramedullary tissues have been shown to significantly influence MK transcriptomic states and functions [ 37 , 49, 50]. The heterogeneity of MKs has long been acknowledged. scRNA sequencing studies in mice have identified three major MK subtypes: platelet-producing MKs (pMKs), niche-supporting MKs (nMKs), and immune MKs (iMKs) [51]. iMKs constitute approximately 5% of total MKs in mouse bone marrow and are characterized by low ploidy and enriched expression of genes involved in inflammatory responses and myeloid leukocyte activation [37]. Interestingly, splenic MKs also exhibit immune-like transcriptional signatures even under steady-state conditions [52], suggesting that extramedullary MKs may be inherently poised for immune function [37]. 

The increased MKs observed in patients with PICS are consistent with previous reports demonstrating an increased presence of MKs under inflammatory and infectious conditions [7]. Our scRNA-seq data revealed that the MK1 and MK2 subsets were predominant in PICS, suggesting an immunoregulatory shift in MK function. Functional enrichment analysis revealed that in these cell subsets, genes associated with antigen processing and presentation, IL-17 signaling, and type I interferon responses are upregulated, whereas genes related to apoptotic pathways are downregulated, thereby promoting the persistence of MKs in the circulation [36]. Notably, as the IL-17 pathway has been implicated in chronic inflammatory conditions, the finding of enriched IL-17 signaling supports the hypothesis that MKs contribute to a sustained immune response in PICS [ 11, 53]. 

The immunophenotype of MKs is plastic, and to some extent, their key immunomodulatory effects are determined by the tissue environment [ 49 , 54]. MKs expanded in all patients, especially in those with PICS, and the immune-related subpopulation MK1 was the most abundant and was considerably more abundant in our study than in a previous report [7]. Circulating and extramedullary MKs originate from the bone marrow; however, inflammatory processes can alter the heterogeneity of bone marrow MKs, leading to an increase in immune-associated MKs [ 37 , 38]. Although no direct lineage tracing has confirmed reversibility between MK1 and MK2, prior studies support the high functional plasticity of MK subpopulations in response to inflammatory stimuli [ 55, 56]. For example, IL-1β, IFN-γ, or TLR signals can upregulate MHC-II and immune modulators in MKs, promoting an MK1-like phenotype [57], whereas IL-6 or TGF-β favors platelet-generating MK2-like features [58]. The inflammatory phase may further influence MK subset dynamics: MK2 predominates during acute infection, supporting coagulation and neutrophil activation, whereas MK1 becomes more prominent during chronic or recovery stages to mediate immune regulation—consistent with our findings in PICS and sepsis. We hypothesize that MK1 and MK2 may represent context-dependent transcriptional states from a shared precursor shaped by cytokines, pathogen-associated molecular patterns, and immune interactions [7]. Future lineage tracing and *in vitro* stimulation studies are needed to clarify this transition. 

Previous studies have shown that various immune cells interact via cytokine secretion and direct ligand-receptor interactions [39]. Our ligand-receptor analysis revealed that MKs actively communicate with monocytes, macrophages, and T, B, and NK cells, suggesting that they may play a broad role in shaping the immune environment in the PICS [11]. These interactions may contribute to the prolonged immune dysfunction observed in patients with PICS, in which excessive immune activation coexists with immunosuppressive mechanisms [10]. The upregulation of antigen processing and presentation pathways suggests that MKs may function as antigen-presenting cells in the PICS microenvironment. The ability of MKs to engage in antigen processing and presentation further supports their emerging role as immune regulators that potentially influence the activation and differentiation of T, B, and NK cells in the context of chronic inflammation. 

Previous studies have shown that MKs express major histocompatibility complex molecules [ 59, 60]. In this study, the MKs in PICs expressed high levels of immune-regulatory surface molecules, including HLA-A, HLA-B, and HLA-C, which engage NK cell inhibitory receptors, and immune checkpoint ligands, such as CD47, which may contribute to immune suppression by interacting with inhibitory receptors on T cells and macrophages [46]. This dual role of MKs in promoting immune activation through antigen presentation, while simultaneously inhibiting excessive immune responses, highlights their complex functions in immune regulation [47]. 

In addition to their interactions with adaptive immune cells, MKs also regulate innate immune responses in PICS. The enrichment of platelet degranulation and neutrophil activation pathways in MK2 subgroup cells suggests that these pathways may enhance the antimicrobial function of neutrophils. Previous studies have shown that MK-derived factors, such as PF4 and CCL5, play crucial roles in neutrophil recruitment and activation [ 61, 62], and our findings suggest that the MKs in PICs may decrease inflammatory states by promoting neutrophil-mediated responses [61]. 

Our functional validation experiments in PICS model mice revealed that the transfer of MKs reduced systemic levels of proinflammatory cytokines and increased chemokine secretion, suggesting a regulatory role for MKs in inflammation [62]. Specifically, the transfer of MKs can reduce the serum levels of TNF-α and IL-17A, two key mediators of systemic inflammation in sepsis and PICS [63]. These findings align with studies showing that MKs can secrete immunosuppressive cytokines such as TGF-β, which modulate inflammatory responses [64]. Additionally, we observed that MKs protected the lungs of PICS mice. Our histological analysis revealed that the injection of MKs reduced alveolar wall damage, despite persistent thrombus formation. This finding aligns with previous reports, suggesting that MKs can migrate to the pulmonary circulation and contribute to tissue repair [46]. The ability of MKs to exert protective effects in the lungs may be mediated by their interactions with macrophages and endothelial cells, thereby dampening excessive inflammation and promoting epithelial regeneration [ 49, 65]. Given the lack of benefit observed with TPO administration, we acknowledge that the dosage and duration of TPO treatment in our current experimental setup may not have been sufficient to elicit a functional response in MKs. Further optimization and mechanistic studies are warranted to fully elucidate the therapeutic potential of TPO in this context. 

The identification of MKs as immunoregulators in PICS opens new avenues for potential therapeutic interventions. Given their dual roles in immune activation and suppression, targeting MK-specific pathways may provide novel strategies for modulating immune dysfunction in survivors of sepsis. Additionally, exploring the potential roles of MK-derived extracellular vesicles in immune modulation could reveal novel biomarkers and therapeutic targets for PICS management [66]. 

Nevertheless, there are several limitations in the current study. First, PICS has various etiologies, including infections, cancer, and trauma [67]. This investigation specifically focused on individuals with sepsis and PICS resulting from gram-negative bacterial infections, thereby maintaining a certain level of homogeneity within the patient cohort. Consequently, caution is advised when these findings are generalized to patients with PICS arising from other conditions (cancer or trauma). Second, patients with PICS were not the same patients who developed PICS after suffering from sepsis. Further studies could achieve more reliable results using samples from the same patient who progresses from sepsis to PICS. Third, we exclusively utilized male mice to eliminate the potential confounding effects of the estrous cycle present in female mice. Fourth, although our data revealed enriched immunoregulatory transcripts and predicted intercellular interactions of MKs in PICs, functional validation of their direct immunosuppressive roles in human immunity through targeted *in vitro* coculture assays or longitudinal human immunophenotyping is needed. 

In conclusion, our study demonstrated that MKs undergo significant expansion and functional reprogramming in the PICS, execute anti-inflammatory activities and regulate both innate and adaptive immunity. The distinct transcriptional signatures of the MK subsets and their interactions with other immune cells suggest their pivotal role in shaping the immune landscape of PICS. These findings provide a foundation for future research into the therapeutic potential of MKs against PICS-induced immune dysfunction.

## Supplementary Data

Supplementary data is available at *Acta Biochimica et Biophysica Sinica* online.
